# Blood substitution—More challenges for translational significance

**DOI:** 10.3389/fstro.2022.1050434

**Published:** 2022-11-09

**Authors:** Xuefang Sophie Ren, Huimahn A. Choi, Aaron M. Gusdon, Heng Hu, Jude Savarraj, Atzhiry Paz, Ryan S. Kitagawa, James W. Simpkins

**Affiliations:** ^1^Department of Neurosurgery, McGovern Medical School, University of Texas Health Science Center at Houston, Houston, TX, United States; ^2^Department of Neuroscience, Center for Basic and Translational Stroke Research, Rockefeller Neuroscience Institute, West Virginia University, Morgantown, WV, United States

**Keywords:** stroke, blood, blood substitution, translation, plasmapheresis

A recent publication described the beneficial effects of whole-blood replacement (BR) in a murine transient middle cerebral artery occlusion model (Ren et al., 2020). We herein emphasize the potential therapeutic importance of this concept and discuss the challenges of developing this strategy into effective treatments for neurological diseases.

The central nervous system (CNS) is a unique, relatively immune-privileged site in which local immune responses are restricted and immunological reactions are relatively limited under normal biological circumstances by the blood-brain barrier (BBB) (Streilein, 1993, 1995; Streilein and Stein-Streilein, 2000). After stroke, the BBB is disrupted which allows antigenic products derived from the brain, such as myelin oligodendrocyte glycoprotein (MOG), myelin basic protein (MBP) and myelin proteolipid protein (PLP), to leak across the damaged BBB into the periphery contributing to the infiltration of peripheral immune cells into the CNS (Lakhan et al., 2009). Moreover, ischemia-induced oxidative stress results in increased production of reactive oxygen species (ROS) in the brain and blood, which causes damage to various biological molecules and cellular components such as DNAs, proteases, and lipids that can be detected in blood following stroke (Drummond and Sobey, 2014; Fisher and Saver, 2015). Stroke causes robust maladaptive changes in blood characterized by a complex systemic/peripheral response. This stroke-induced blood dyscrasia leads to a vicious cycle which further impairs brain cells and worsens outcomes after stroke (Figure 1). The study took a new approach to translational stroke research, abandoning the usual “brain-centric” view in favor of an integrated investigation of evolving brain injury and the blood dyscrasia caused by stroke.

**Figure 1:**
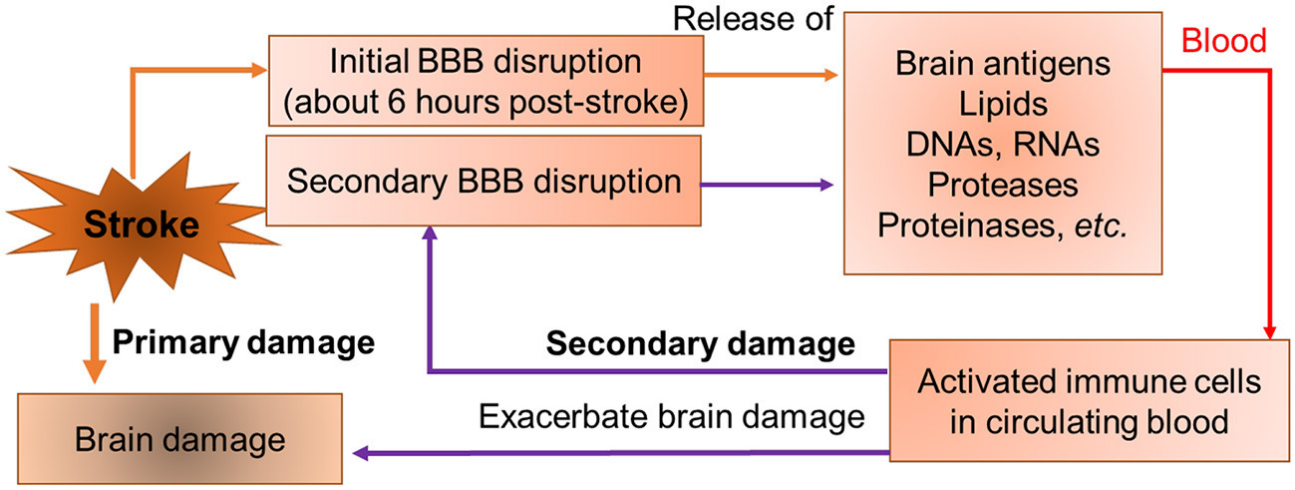
A conceptual model of a vicious cycle in stroke. Stroke causes initial BBB opening at 6 h post-stroke. Brain-specific antigens (such as myelin basic protein *etc*.), matrix metalloproteinases (MMPs), proteases (cathepsins), and necrotic cellular components such as DNAs, RNAs, and lipids are released into peripheral blood after BBB opening. The brain sourced components activate peripheral immune cells in blood. Activated immune cells secrete cytokines and MMPs, produce secondary BBB disruption, and exacerbate outcomes.

The blood substitution treatment reduces stroke progression by removing post-stroke blood and replacing it with blood from normal animals. Both the removal of post-stroke blood and the replacement with normal blood is essential for the beneficial effects. Blood withdrawal removes the deleterious signals produced by stroke, while whole-blood replacement returns beneficial elements seen in normal blood. Although this strategy is feasible in stroke models, whole-blood exchange transfusion is limited in patients because of blood compatibility and the major histocompatibility complex (MHC) from human leukocyte antigen (HLA) that can cause serious adverse reactions to transfusion. Though, it has shown prospective compatible crossmatches on several patients using organs from pigs genetically engineered for human xenotranplantation, which porcine MHC class I and three xenoreactive antigen genes GGTA1, CMAH and B4GALNT2 are knockout (Fischer et al., 2020; Porrett et al., 2022; Pullen, 2022). The large volume of whole-blood replacement needed makes it impractical for direct translation into humans until blood is available from genetically modified large animals. These practical limitations make this type of blood replacement strategy unlikely to be directly translated into human studies currently.

An increasing number of studies show that aging is associated with the functional decline of all tissues and a striking increase in many diseases (Mahmoudi et al., 2019). Emerging rejuvenations have turned back to promising therapeutic strategies. Numerous heterochronic parabiosis experiments in the preclinical mouse model showed that blood from young mice reversed the established aging patterns on cellular, gene expression, and pathway levels (Palovics et al., 2022). These studies could be strong evidence to support the findings in this stroke model. They also highlighted the potential of blood substitution in more complicated conditions, including different diseases and aging, which could be of interest to a broader audience.

Due to the limitations of blood substitution highlighted in the article, it isn't easy to be translated into clinical therapeutics. Plasma exchange, also known as plasmapheresis or dialysis, is used routinely to treat critical patients with autoimmune disorders and hematological diseases (Daga Ruiz et al., 2017; Clark and Huang, 2019; Fernandez-Zarzoso et al., 2019). This treatment can effectively remove pathogenic circulating molecules, antibodies, inflammatory cytokines, adhesion molecules, *etc*. in blood (Reeves and Winters, 2014). Plasmapheresis can be used to replace plasma without removal of blood cells or it can be modified to add additional blood cellular components as required. In order to further develop a translatable strategy for treating stroke patients, we expect that a plasmapheresis strategy could be able to mirror the benefits seen in the blood substitution experiments to treat stroke-induced blood dyscrasia. However, would it be reasonable to expect similar beneficial effects of dialysis on stroke? In these blood substitution studies, a lot of detrimental factors were identified during stroke, which could potentially be removed by dialysis or neutralization antibodies. Further studies focusing on these specific detrimental factors will provide alternatives for translating the blood substitution strategy. On the other hand, further identification of the beneficial cells or proteins/metabolomics in the healthy blood will also help to translate the blood substitution to the clinic.

The published blood substitution strategy has demonstrated that removing blood from stroke mice and replacing it with plasma from healthy mice does not significantly improve outcomes after stroke (Ren et al., 2020). There could be several reasons that cause the failure of plasma transfusion alone in stroke mice. First, BR treatment removes not only detrimental molecules in blood but also protective cells in blood. Second, transfusion of plasma does not return any cellular components that may contain effectors protecting against stroke induced brain damage back to recipient stroke mice. Third, the volume used for plasma transfusion is relatively small as transfusion of 500 μl of plasma is considered < 20% of total blood volume in a mouse and this dose is not sufficient to provide a possible dilution effect. In addition, a recent study demonstrated that plasma from healthy donors protects BBB integrity via FGF21 and improves the recovery in a mouse model of stroke (Mamtilahun et al., 2021). Thus, the data from plasma transfusion should not dampen our enthusiasms for developing plasmapheresis as a treatment modality for stroke.

The majority of stroke patients are unable to receive thrombectomy leading to strokes from permanent arterial occlusions. The current blood substitution strategy has only been evaluated in transient occlusion models and has not been evaluated in permanent stroke models. Additionally, models in stroke animals with stroke risk factors including advanced aging, high blood pressure, diabetes, heart and blood vessel diseases, high LDL cholesterol levels, and viral or bacterial Infections that cause inflammation, need to be studied.

A blood substitution approach may also be a viable strategy in other acute neurological diseases, traumatic brain injury, and hemorrhagic stroke, which show robust systemic responses leading to worse clinical outcomes (Bortolotti et al., 2018; Sweeney et al., 2018; Saand et al., 2019). Once a translatable blood substitution paradigm is experimentally validated in stroke it can easily be studied in other systemic diseases and moved to clinical treatment studies in the near future.

## Author contributions

XSR conceived and wrote the manuscript. HAC, AMG, HH, JS, AP, RSK, and JWS discussed and edited the manuscript. All authors contributed to the article and approved the submitted version.

## Funding

This study is supported by NSF (1916894 to XSR) and UTHealth new faculty start-up (to XSR).

## Conflict of interest

The authors declare that the research was conducted in the absence of any commercial or financial relationships that could be construed as a potential conflict of interest.

## Publisher's note

All claims expressed in this article are solely those of the authors and do not necessarily represent those of their affiliated organizations, or those of the publisher, the editors and the reviewers. Any product that may be evaluated in this article, or claim that may be made by its manufacturer, is not guaranteed or endorsed by the publisher.
